# Predictive modeling of structured electronic health records for adverse drug event detection

**DOI:** 10.1186/1472-6947-15-S4-S1

**Published:** 2015-11-25

**Authors:** Jing Zhao, Aron Henriksson, Lars Asker, Henrik Boström

**Affiliations:** 1 Department of Computer and Systems Sciences, Stockholm University, Borgarfjordsgatan 12, SE-16407 Kista, Sweden

**Keywords:** pharmacovigilance, adverse drug events, electronic health records, machine learning, random forest, feature selection

## Abstract

**Background:**

The digitization of healthcare data, resulting from the increasingly widespread adoption of electronic health records, has greatly facilitated its analysis by computational methods and thereby enabled large-scale secondary use thereof. This can be exploited to support public health activities such as pharmacovigilance, wherein the safety of drugs is monitored to inform regulatory decisions about sustained use. To that end, electronic health records have emerged as a potentially valuable data source, providing access to longitudinal observations of patient treatment and drug use. A nascent line of research concerns predictive modeling of healthcare data for the automatic detection of adverse drug events, which presents its own set of challenges: it is not yet clear how to represent the heterogeneous data types in a manner conducive to learning high-performing machine learning models.

**Methods:**

Datasets from an electronic health record database are used for learning predictive models with the purpose of detecting adverse drug events. The use and representation of two data types, as well as their combination, are studied: clinical codes, describing prescribed drugs and assigned diagnoses, and measurements. Feature selection is conducted on the various types of data to reduce dimensionality and sparsity, while allowing for an in-depth feature analysis of the usefulness of each data type and representation.

**Results:**

Within each data type, combining multiple representations yields better predictive performance compared to using any single representation. The use of clinical codes for adverse drug event detection significantly outperforms the use of measurements; however, there is no significant difference over datasets between using only clinical codes and their combination with measurements. For certain adverse drug events, the combination does, however, outperform using only clinical codes. Feature selection leads to increased predictive performance for both data types, in isolation and combined.

**Conclusions:**

We have demonstrated how machine learning can be applied to electronic health records for the purpose of detecting adverse drug events and proposed solutions to some of the challenges this presents, including how to represent the various data types. Overall, clinical codes are more useful than measurements and, in specific cases, it is beneficial to combine the two.

## Background

With the adoption of computerized medication ordering and administration systems, the veil on the incidence of adverse drug events (ADEs) is slowly being removed. Unfortunately, ADEs are still considered to be heavily under-reported [1]. Among the ADEs that are reported, around half are preventable [2], causing unnecessary suffering for patients and increased healthcare costs. According to one meta-analysis, ADEs are, in fact, responsible for around 4.9% of hospital admissions worldwide, and, in some cases, this number can be as high as 41.3% [3]. There is thus no doubt that drug safety is an important public health problem. Unfortunately, the high rate of ADEs may continue unabated unless systems that provide decision support for drug selection and dosing are developed and more widely implemented at the point of care [4].

### Pharmacovigilance using electronic health records

Efforts have been made in pharmacovigilance to improve drug safety. The World Health Organization (WHO) defines pharmacovigilance as "the science and activities relating to the detection, assessment, understanding and prevention of adverse effects or any other drug-related problem" [5]. The primary resources involved in pharmacovigilance are clinical trials, spontaneous reports and longitudinal healthcare databases [6]. The use of these can be divided into pre-marketing and post-marketing pharmacovigilance activities. In the pre-marketing stage, prior to the launch of a drug, clinical trials are used to gather information on both the efficacy and safety of a drug. However, such a source of information comes with two inherent limitations, namely small samples of participants and short study duration. These limitations make it challenging to identify ADEs that are rare or occur with a long latency. In the post-marketing stage, after the drug has been launched, spontaneous reporting systems are used continuously to collect information on the safety of the drug. Examples of such systems are the US Food and Drug Administration's Adverse Event Reporting System [7] and WHO's Global Individual Case Safety Reports Database, Vigibase [8]. Spontaneous reports are voluntarily made by patients and physicians of suspected ADEs, which allows for monitoring of all drugs on the market at a fairly low cost. Unfortunately, such systems suffer heavily from under-reporting: it has been estimated that more than 94% of ADEs are not reported through spontaneous reports [9]. Other limitations of spontaneous reports include selective reporting, incomplete patient information and indeterminate population information; for more details see [10]. Indeterminate population information is particularly problematic since it prevents the calculation of the incidence of reported ADEs. As a result of these limitations, the need for alternative, complementary data sources is duly being acknowledged.

Among the possible alternative data sources, which also includes social media and medical literature, are electronic health records (EHRs) [11] since they capture and integrate patient data from all aspects of clinical observations over time. Although the main function of EHRs is to archive and manage patient data efficiently - in comparison to paper-based health record systems - secondary use of EHR data is currently being widely explored for various medical research, such as disease discovery and patient stratification [12,13], among which also pharmacovigilance has received a lot of attention. There are various ways of utilizing EHRs for pharmacovigilance in a data-driven fashion, such as calculating correlations between drugs and diseases, clustering patients into different disease groups, and employing machine learning based prediction [14], among which the latter is particularly nascent.

### Predictive modeling of data from electronic health records

Machine learning based methods are data-driven approaches that can support discovery and exploitation of statistical patterns from large quantities of data. Given a large amount of observations that are described by multiple variables, such methods have proven to be robust to random errors [15]. In areas where there is a need to analyze large amounts of data, such as bioinformatics, machine learning is a key technique, particularly when analyzing "big data" [16]. This is also the case in post-marketing drug safety surveillance, where the discovery process typically relies on large samples; computational signal detection algorithms have in this context been developed to analyze data with the purpose of detecting signals of potential ADEs [17]. Some of these algorithms detect signals according to a score function based on contingency tables, such as disproportionality analysis of spontaneous reports. However, a limitation of using contingency tables is that, by reducing the analysis to only two dimensions, the potential concomitant loss of clinically crucial information may result in arbitrary associations [17,18]. This can be eschewed by instead employing multivariate algorithms for signal detection, where machine learning methods can provide efficient and effective means of modeling high-dimensional data.

**Figure 1:**
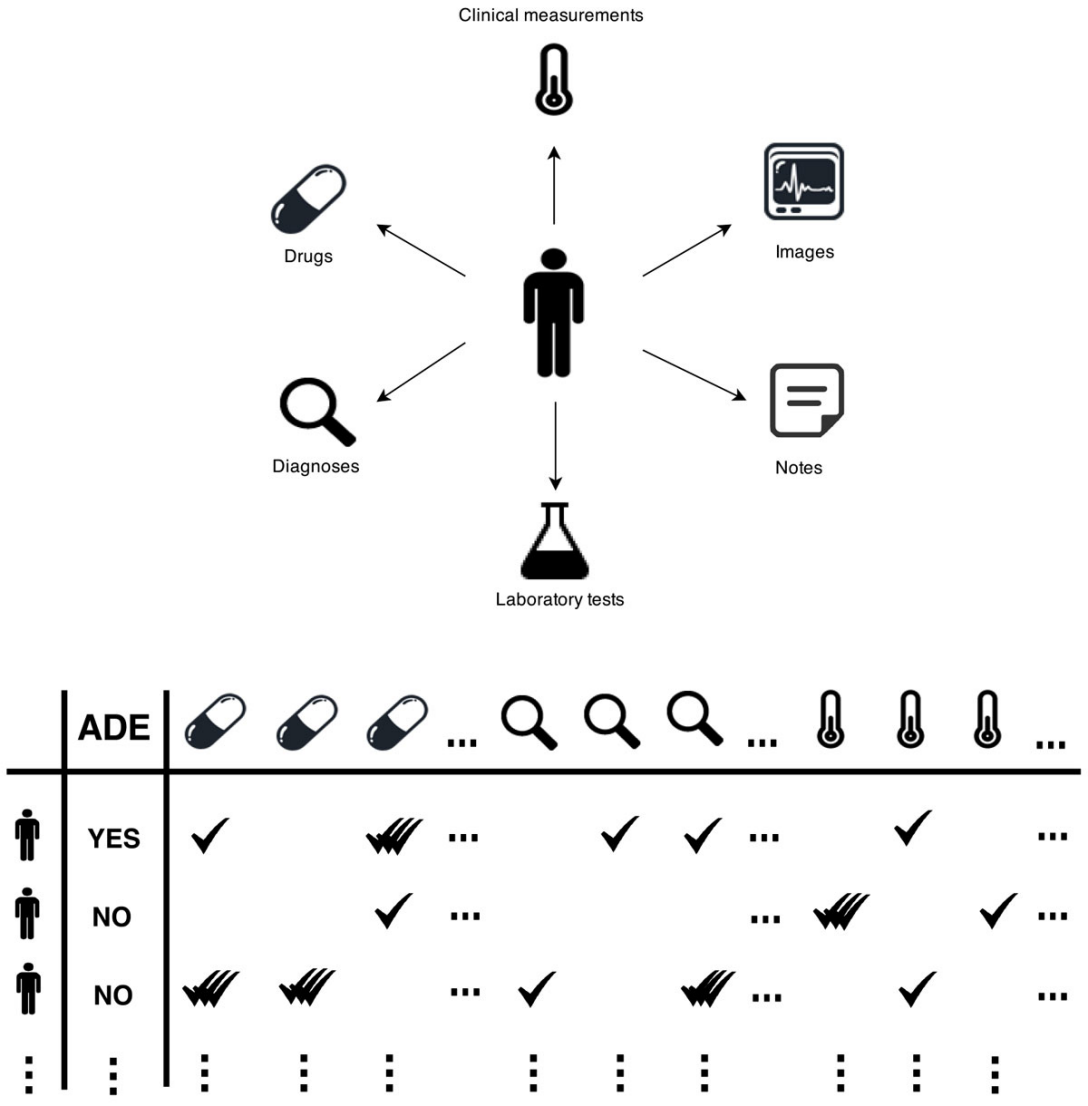
**Extracting data for machine learning methods from electronic health records**.

Applying machine learning to EHR data is, however, challenging for various reasons. A natural way of fitting EHR data into machine learning models is to utilize the various clinical events that are recorded in EHRs as variables to describe, for instance, patients. For each patient, these clinical events can be represented either as a sequence according to reporting chronology, or as a bag, in effect discarding order information. Treating clinical events as sequences is, however, problematic for two reasons: (1) many events have identical timestamps, which raises the question of how to deal with simultaneously occurring events; (2) there is a lack of understanding to what extent the order of reported events reflects reality, i.e., we cannot know whether the sequence of reported events is the same as the actual sequence of events. When representing clinical events as a bag, there are other problems that need to be handled, as illustrated in Figure 1. On the one hand, the data is often high-dimensional and sparse, i.e., a large number of features describe each patient, but many features have non-zero values only for a small fraction of the patients. On the other hand, the types of data available in EHRs are heterogeneous and complex. Typically, EHR data includes both structured data according to predefined templates, such as demographic patient information, drug prescriptions, diagnoses, clinical measurements and lab tests, as well unstructured data in the form of clinical notes written in natural language. Moreover, for some types of data, such as prescribed drugs, assigned diagnoses and obtained clinical measurements, a patient may have experienced the same type of clinical event multiple times, for instance a patient being prescribed a certain drug multiple times. In summary, the challenges of analyzing EHR data with machine learning methods stem not only from high dimensionality and sparsity, but also from the existence of different data types that are tangled together with missing and duplicated values.

### Related work

Due to limited access to EHR data, research on exploiting it for pharmacovigilance is still relatively scarce compared to using other data sources, despite its acknowledged potential. Among the published research on using EHRs for ADE detection, some have focused on using clinical notes [19-21], while how best to exploit the structured data remains under-explored. In some studies, however, clinical measurements or lab tests from EHRs have been utilized for (adverse) event detection by representing them as time series [22], aggregating them into categorical variables [23], or representing them from multiple perspectives [24]. Other studies have used diagnoses and drugs instead [25,26], while these data types have also been used in conjunction for signaling ADEs, albeit only in a case study and on a very limited scale [27].

Diagnoses and drugs are normally encoded by standard coding systems such as International Statistical Classification of Diseases and Related Health Problems (ICD) and Anatomical Therapeutic Chemical Classification System (ATC), respectively. These coding systems have their own concept hierarchies representing terms from general levels to more specific ones according to organ system or etiology. In a previous study, we have studied the possibility of exploiting these concept hierarchies to obtain improved predictive performance on the task of distinguishing between patients who have experienced a specific ADE and randomly selected patients who have not experienced that same ADE [26]. It was shown that for such tasks, using only the more general levels of the codes is sufficient to maintain the predictive performance on a high level. We have also evaluated various ways of representing clinical measurements from EHRs and discovered that using such measurements alone still leads to the effective detection of ADEs; moreover, using only the number of times each clinical measurement has been taken, without considering their actual values, is a representation that results in the highest predictive performance for the most common learning algorithms [24].

However, previous studies have either used a single data type from EHRs or a small number of pre-selected variables from different data types to signal a specific ADE. In this study, we explore if it is beneficial to combine various data types, on a large scale, by using all of the available variables for ADE detection, and also how best to represent them. In addition to detecting specific ADEs, this study aims to explore ways of using structured EHR data that can be exploited to detect a wide range of ADEs, which could be adopted in a general decision support system that alerts for potential ADEs.

## Methods

In this study, we investigated the use of various data types in EHRs for drug safety surveillance. Here, we focused on using the structured data to build predictive models using machine learning based methods. Clinical measurements, diagnoses and drugs were extracted from a real EHR database. Besides the known problems of EHR data such as high dimensionality and sparsity, these data types have their own characteristics and hence lead to different challenges when fitting them into predictive models. For example, some clinical events here might be observed multiple times for one patient, while some might not be observed at all. Therefore, a series of experiments were conducted to explore the use of these heterogeneous data types separately and together when predicting ADEs with machine learning based methods: first, different representations of each data type were compared and the best representation of the corresponding data type was selected for merging with the other data types, i.e., to form a fused feature set, which was compared to using each data type separately; second, to reduce the high dimensionality and sparsity, feature selection was conducted on both the separate data types and the fused feature set, which also allowed for an in-depth feature analysis; and finally, various commonly used learning algorithms were applied and compared for the classification task.

### Data source

Data was extracted from a Swedish EHR database, the Stockholm EPR Corpus (this research has been approved by the Regional Ethical Review Board in Stockholm with permission number 2012/834-31/5). This database contains health records of around 700,000 patients from 2009 to 2010, which were obtained from Karolinska University Hospital in Stockholm, Sweden [28]. Here, large amounts of diagnosis information, drug administrations, clinical measurements, lab tests and clinical notes in free-text from anonymized health records are available for research. In this study, we only extracted the structured data, i.e., diagnoses, drugs and clinical measurements.

In the Stockholm EPR Corpus, diagnoses are encoded by the International Statistical Classification of Diseases and Related Health Problems, 10th Edition (ICD-10), some of which indicate ADEs, e.g., G44.4 (drug-induced headache). To create training data for building machine learning models, we used these ADE-related diagnosis codes as class labels. The population is hence divided into patients that have been assigned an ADE-related diagnosis code and those who have not. In a study on the use of ICD-10 codes for ADE reporting [29], the ADE-related diagnosis codes were divided into categories according to the strength of their indication for ADEs, where category A.1 (a drug-related causation was noted in the diagnosis code) and category A.2 (a drug-or other substance-related causation was noted in the diagnosis code) were used in this study, as they indicate the most certain causal drug-diagnosis relationship of ADEs compared to the other categories.

To avoid spurious findings, we have selected 27 ADE-related codes that are most frequently used in the Stockholm EPR Corpus, resulting in 27 datasets, where the existence of each ADE-related diagnosis code indicating a particular ADE served as the class label in each dataset; see Table 1 for the selected ADE-related diagnosis codes and their description. The classification task is hence binary: positive or negative with respect to a specific ADE. In each dataset, examples correspond to patients: patients whom have been assigned an ADE-specific diagnosis code constitute positive examples and patients whom have been assigned a *similar *diagnosis code to the ADE-specific diagnosis code form negative examples, where two codes are considered *similar *if they share the first three levels in the concept hierarchy. For instance, if the positive examples are patients with diagnosis code G44.4 (drug-induced headache), the negative examples are patients with any diagnosis code starting with G44 (other headache syndromes), but not G44.4. Features are clinical events, i.e., diagnoses, drugs and clinical measurements, that are reported in the health records of these patients prior to the event of interest, i.e., the class label. The number of instances, the proportion of the positive class and the number of features from each data type for each dataset are described in Table 2.

**Table 1:** The 27 selected ADE related diagnosis codes.

Code	Description
D642	Secondary sideroblastic anemia due to drugs and toxins
E273	Drug-induced adrenocortical insufficiency
F110	Mental and behavioural disorders (MBDs) due to use of opioids: acute intoxication
F112	MBDs due to use of opioids: dependence syndrome
F130	MBDs due to use of sedatives or hypnotics: acute intoxication
F132	MBDs due to use of sedatives or hypnotics: dependence syndrome
F150	MBDs due to use of other stimulants, including caffeine: acute intoxication
F151	MBDs due to use of other stimulants, including caffeine: harmful use
F152	MBDs due to use of other stimulants, including caffeine: dependence syndrome
F190	MBDs due to multiple drug use: acute intoxication
F192	MBDs due to multiple drug use: dependence syndrome
F199	MBDs due to multiple drug use: unspecified mental and behavioural disorder
G240	Drug-induced dystonia
G251	Drug-induced tremor
G444	Drug-induced headache, not elsewhere classified
G620	Drug-induced polyneuropathy
I427	Cardiomyopathy due to drugs and other external agents
I952	Hypotension due to drugs
L270	Generalized skin eruption due to drugs and medicaments
L271	Localized skin eruption due to drugs and medicaments
O355	Maternal care for (suspected) damage to fetus by drugs
T782	Adverse effects: anaphylactic shock, unspecified
T783	Adverse effects: angioneurotic oedema
T784	Adverse effects: allergy, unspecified
T808	Other complications following infusion, transfusion and therapeutic injection
T886	Anaphylactic shock due to correct drug or medicament properly administered
T887	Unspecified adverse effect of drug or medicament

**Table 2:** Statistical description of 27 datasets.

			Number of features		
			
Dataset	Instances	**% Pos**.	Codes	Measurements	Combination
D642	3733	2.87%	3999	494	8262
E273	183	12%	912	240	2935
F110	146	22.6%	1051	205	2958
F112	146	63.7%	1054	205	2963
F130	112	54.5%	779	142	2237
F132	112	27.7%	777	142	2231
F150	111	14.4%	476	107	1543
F151	111	17.1%	475	107	1542
F152	111	69.4%	481	111	1573
F190	168	31.5%	869	160	2454
F192	168	50%	865	160	2447
F199	168	8.93%	866	160	2448
G240	68	20.6%	444	136	1636
G251	194	6.7%	1014	263	3209
G444	908	2.5%	1774	318	4594
G620	382	6%	1624	280	4152
I427	448	5.1%	1341	299	3852
I952	483	8.3%	1654	333	4471
L270	435	35.9%	1297	325	3912
L271	434	11.1%	1286	325	3897
O355	237	35.4%	736	110	1930
T782	1203	8.5%	1625	319	4405
T783	1207	8.6%	1627	319	4408
T784	1213	60.8%	1628	319	4409
T808	391	87.5%	1229	271	3533
T886	715	6.2%	2226	401	5606
T887	716	61.7%	2230	400	5604

### Experimental setup

The main underlying learning algorithm in this study is random forest [30], which is an ensemble learning method that generates a set of decision trees. Each tree in the forest is built with a bootstrapped sample from the original training examples and each node in the tree only considers a randomly selected subset of the original feature set. The trees carry out the learning task independently from each other and the forest eventually outputs the final result through voting, i.e., averaging the output of all constituent trees. The random forest learning algorithm has become one of the most popular machine learning methods, especially in bioinformatics where data is often high dimensional, as a result of its relatively low computational cost and robust predictive performance [31].

Evaluation was done through 10-fold cross validation with 10 iterations. The performance metrics used in this study are accuracy and area under ROC curve (AUC). Accuracy, the most common and perhaps also the most intuitive metric to evaluate the performance of a predictive model, measures the percentage of examples that are predicted correctly. Area under ROC curve can be used whenever the learning algorithm is able to rank the examples based on the decreasing probability of predicting them as positive. It measures the probability of ranking a true positive example ahead of a false positive example [32], i.e., the rate of detecting true signals versus the false alarm rate. Compared to accuracy, AUC is sometimes favored because it is not sensitive to changes in the class distribution between training and test data.

When more than two models were compared, a Friedman test [33] was employed to test the statistical significance, where the rank of each model is used. To look further at the pairwise significance between the inspected models, a post-hoc test using the Bergman-Hommel procedure was applied [34].

#### Using various data types

In the first experiment, different representations of clinical measurements, on the one hand, and diagnoses and drugs on the other (here we consider diagnoses and drugs as one data type, namely *clinical codes*, as they share the same characteristics), as well as their combination, were compared.

##### Clinical measurements

In a previous study [24], we proposed five representations (listed below) of clinical measurements to handle the problem that each measurement can be observed multiple times for a patient. Here, we re-evaluated the use of these representations, as well as their combination, on a slightly different task.

• **Mean **- the average of the observed values

• **SD **- the standard deviation of the observed values

• **Slope **- the difference between the first and last observation over the time span

• **Existence **- whether or not a measurement has been taken

• **Count **- the number of times a measurement was taken

##### Clinical codes

**Figure 2:**
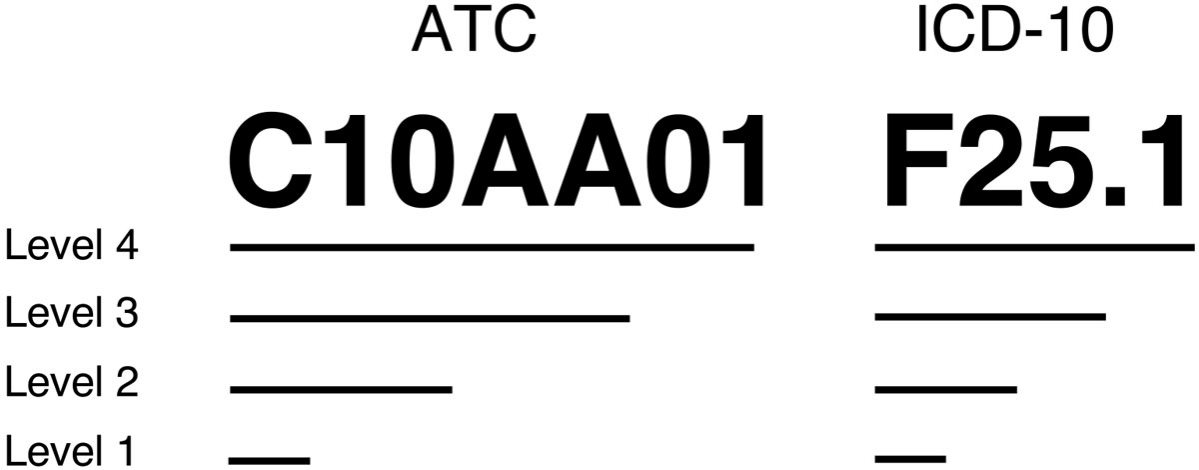
**Concept hierarchies of ATC and ICD-10 codes**. C10AA01 is the ATC code for Simvastatin and F25.1 is the ICD-10 code for Schizoaffective disorder.

Diagnoses are encoded by the ICD-10 system and drugs by the ATC system in the Stockholm EPR Corpus, both of which have inherent concept hierarchies that can be used to aggregate the clinical codes into different hierarchical levels, as shown in Figure 2. Here, we compared using the different levels of clinical codes to a combination of all levels.

After investigating representations of clinical measurements and clinical codes separately, we combined them using their respective best observed representation. As it has previously been shown that, when an ensemble model is employed, building the model from a fused set of data types is favored compared to fusing ensemble models built from the individual data type [35], we combined the two data types by fusing them into one feature set before applying the random forest algorithm. The predictive performance of random forests using clinical measurements, clinical codes and a combination of the two were compared.

#### Feature selection

In a follow-up experiment, feature selection was added to the pipeline prior to building the predictive models in order to remove those features that are not informative, while simultaneously reducing the dimensionality and, in some cases, sparsity. There are two common types of feature selection approaches: wrapper-based and filter-based. The former utilizes the targeted learning algorithm as a black box to evaluate the usefulness of features according to their predictive performance [36], while the latter selects features according to a score function independent of the chosen learning algorithm [37]. Wrapper-based approaches are generally considered to produce better feature subsets but with much higher computational costs compared to the filter-based approaches. In this study, we used a filter-based approach to univariate feature selection, information gain, to select relatively important features, where the features are first ranked according to the information gain between them and the class label before selecting the top-ranked ones. The information gain for a certain feature is calculated as the difference between the entropy before splitting the training examples with this feature and the entropy after splitting. The entropy of the random variable *x *is

$$H\left(x\right)=-\underset{x}{\sum }p\left(x\right){log}_{2}p\left(x\right),$$

where *p*(*x*) is the probability distribution of *x*. In this case, the entropy before splitting is the entropy by splitting the examples only according to the class label *Y *, *H*(*Y*); and the entropy after splitting the examples on feature *f *is

$$H\left(Y|f\right)=\underset{f,Y}{\sum }p\left(Y\right)H\left(Y_{f}\right),$$

where, *Y_f _*is the probability distribution of the class label given feature *f*. Therefore, the information gain of feature *f *is

I(f,Y)=H(Y)-H(Y|f).

In this study, we explored the impact of feature selection on the predictive performance of the random forest algorithm with a set of thresholds starting from the top 10% of available features ranked according to their information gain scores and subsequently adding an extra 10% until the full feature set is included.

#### Using various learning algorithms

The random forest algorithm is known for being robust with high dimensional data; therefore, in the last experiment, eight additional commonly used learning algorithms were applied in order to find out if the observation from using random forest holds for the others and also to study the impact of feature selection on this task. The selected learning algorithms and their parameters are listed in Table 3. Each learning algorithm used clinical measurements and clinical codes, in isolation and combined, to build predictive models with features selected on all thresholds.

**Table 3:** Learning algorithms and their default settings.

Classifier	Description	Notes
DT	CART decision tree	minimum 1 instance per leaf
SVM Poly	Support Vector Machine	polynomial kernel of degree 3
SVM RBF	Support Vector Machine	RBF kernel, gamma = 0.0
LogReg	Logistic Regression	L2 regularization
kNN	k nearest neighbors	k = 5
AdaBoost	Adaptive boosting	Decision trees, 50 base estimators
Bagging	Bagging using CART tree	10 base estimators
NB	Naïve Bayes	
RF	Random forest	500 trees, inspected features = $\sqrt{n\;}$

## Results

In this section, we report on the predictive performance, in terms of accuracy and AUC, of models generated with the random forest algorithm that was provided with various representations of 27 clinical datasets, each one containing a different data type (clinical codes and measurements) and representation, as well as combinations of these - with and without feature selection. We present both results from individual datasets, as well as summary results, averaged over datasets. An in-depth feature analysis is moreover conducted and, finally, results from using various learning algorithms are summarized.

### Using various data types

The clinical measurements were represented in five distinct ways - Mean, SD, Slope, Existence and Count - as well as a combination of these. Accuracy and AUC, averaged over 27 datasets, as obtained by random forest models with access to the each of the representations are presented in Table 4. For both accuracy and AUC, using the combined representation yielded the best performance. The clinical codes, on the other hand, were aggregated - save for the most specific level - into more general levels according to their concept hierarchies. The averaged accuracy and AUC of random forests with access either to a single level or a combination of all four levels are shown in Table 5 from which we can see that the predictive performance was improved when including all levels of the concept hierarchies.

**Table 4:** Comparing multiple representations of clinical measurements.

	Accuracy (rank)	AUC (rank)
Mean	80.75 (2.96)	0.635 (2.74)
SD	80.23 (3.44)	0.535 (5.33)
Slope	80.54 (3.33)	0.612 (3.52)
Existence	79.25 (4.48)	0.604 (4.26)
Count	80.54 (3.63)	0.633 (2.96)
All	81.41 (2.74)	0.655 (2.19)

P-value	0.01	<0.0001

**Table 5:** Comparing different levels of clinical codes.

	Accuracy (rank)	AUC (rank)
Level 1	83.24 (3.37)	0.731 (3.74)
Level 2	84.08 (2.78)	0.742 (3.41)
Level 3	83.80 (2.93)	0.757 (2.81)
Level 4	83.93 (2.67)	0.763 (2.67)
All	84.47 (2.44)	0.763 (2.37)

P-value	0.17	0.008

A random forest provided with a fused feature set, comprising the best representations of clinical measurements and clinical codes, was then built and compared with random forests with access only to one of the data types. The number of features of the fused feature set is presented in Table 2 under *Combination*. The accuracy and AUC for the 27 datasets are listed in Table 6. According to a Friedman test, the observed differences among the three random forests is significant, in terms of both accuracy and AUC, and the post-hoc analysis indicates that only using clinical measurements leads to significantly worse predictive performance compared to using clinical codes and their combination; however, there is no significant difference between the latter two.

**Table 6:** Comparing random forests using clinical measurements (M), clinical codes (C) and their combination (M+C).

	Accuracy			AUC		
		
Dataset	M	C	M+C	M	C	M+C
D642	98.79 (3)	98.95 (2)	99.03 (1)	0.961 (3)	0.980 (2)	0.994 (1)
E273	86.98 (3)	87.51 (1)	87.51 (1)	0.691 (3)	0.706 (2)	0.741 (1)
F110	80.45 (2)	83.14 (1)	80.38 (3)	0.676 (3)	0.824 (1)	0.798 (2)
F112	68.48 (2)	72.73 (1)	66.30 (3)	0.672 (3)	0.803 (1)	0.752 (2)
F130	54.97 (3)	60.61 (1)	56.89 (2)	0.573 (3)	0.666 (1)	0.646 (2)
F132	71.33 (1)	69.47 (3)	69.47 (2)	0.558 (3)	0.686 (1)	0.616 (2)
F150	84.02 (3)	86.85 (1)	85.85 (2)	0.706 (3)	0.901 (1)	0.885 (2)
F151	84.68 (1)	82.03 (2)	82.03 (2)	0.502 (3)	0.619 (1)	0.535 (2)
F152	72.82 (3)	76.30 (1)	74.95 (2)	0.733 (3)	0.838 (1)	0.826 (2)
F190	64.78 (3)	74.58 (1)	72.88 (2)	0.608 (3)	0.805 (1)	0.782 (2)
F192	60.07 (3)	67.33 (1)	61.05 (2)	0.660 (3)	0.730 (1)	0.682 (2)
F199	90.04 (3)	91.61 (1)	90.98 (2)	0.568 (3)	0.577 (2)	0.700 (1)
G240	78.33 (3)	81.31 (1)	81.31 (1)	0.596 (3)	0.622 (2)	0.639 (1)
G251	93.34 (1)	93.29 (2)	93.29 (2)	0.328 (3)	0.719 (1)	0.523 (2)
G444	97.47 (3)	97.51 (1)	97.51 (1)	0.479 (3)	0.631 (2)	0.666 (1)
G620	93.47 (3)	94.26 (1)	94.26 (1)	0.509 (3)	0.765 (1)	0.756 (2)
I427	95.77 (3)	96.57 (2)	96.80 (1)	0.713 (3)	0.895 (1)	0.891 (2)
I952	91.92 (1)	91.63 (3)	91.84 (2)	0.517 (3)	0.552 (1)	0.542 (2)
L270	86.65 (1)	85.20 (3)	85.70 (2)	0.909 (2)	0.908 (3)	0.915 (1)
L271	89.17 (2)	89.84 (1)	89.10 (3)	0.784 (3)	0.800 (2)	0.802 (1)
O355	62.00 (3)	90.96 (2)	91.43 (1)	0.642 (3)	0.962 (1)	0.956 (2)
T782	91.02 (3)	91.90 (2)	92.09 (1)	0.695 (3)	0.712 (2)	0.717 (1)
T783	90.39 (3)	91.27 (1)	91.18 (2)	0.774 (3)	0.845 (2)	0.862 (1)
T784	60.44 (3)	68.63 (2)	68.82 (1)	0.611 (3)	0.732 (2)	0.753 (1)
T808	86.45 (3)	93.88 (1)	91.59 (2)	0.857 (3)	0.953 (2)	0.962 (1)
T886	93.57 (3)	94.05 (1)	94.05 (1)	0.629 (3)	0.655 (2)	0.656 (1)
T887	70.65 (2)	69.24 (3)	70.94 (1)	0.721 (2)	0.720 (3)	0.754 (1)

Average	81.41 (2.48)	84.47 (1.56)	83.6 (1.70)	0.655 (2.93)	0.763 (1.56)	0.754 (1.52)
		
P-value	0.007			< 0.0001		

### Using the most informative features

**Figure 3:**
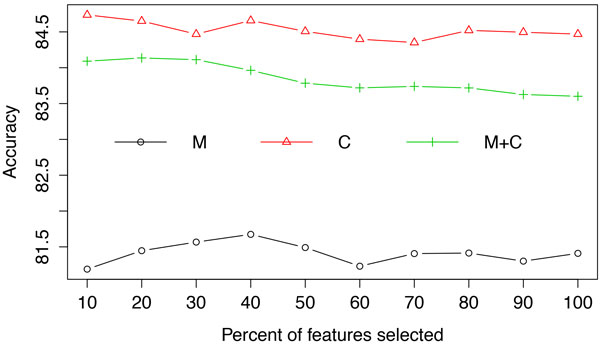
**Averaged accuracy from random forests using clinical measurements (M), clinical codes (C) and their combination (M+C) at each feature selection threshold**.

**Figure 4:**
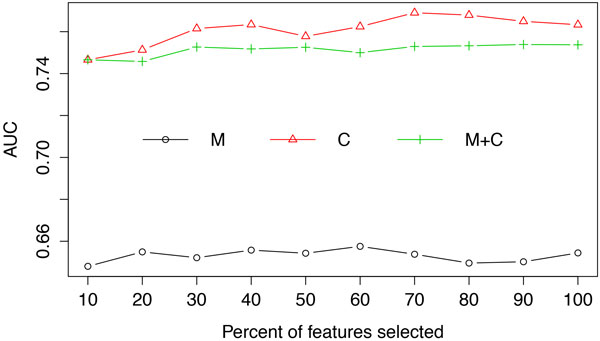
**Averaged AUC from random forests using clinical measurements (M), clinical codes (C) and their combination (M+C) at each feature selection threshold**.

The performance of random forests using clinical measurements, clinical codes and their combination after selecting different proportions of the most informative features according to their information gain scores are shown in Figure 3 for accuracy and Figure 4 for AUC. From these results we can see that applying feature selection improved the predictive performance, albeit on a small scale. However, even when employing feature selection, the addition of clinical measurements fails to improve the predictive performance compared to using only clinical codes. An explanation for this can be sought by investigating the outcome from different perspectives.

**Figure 5:**
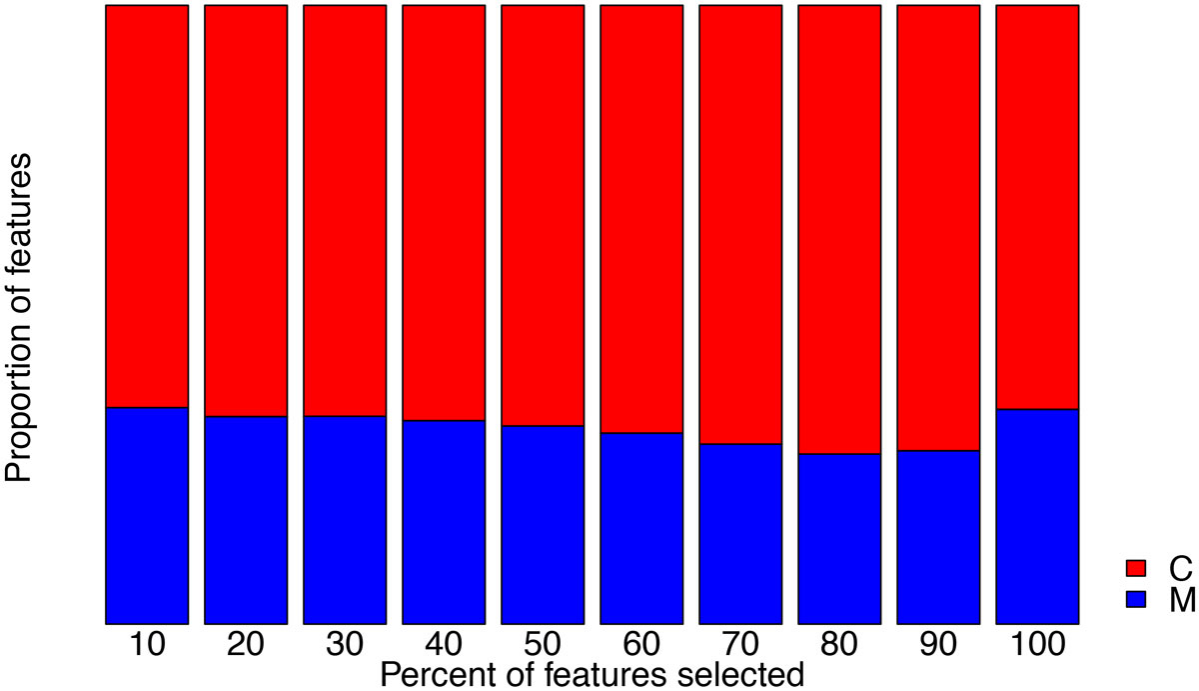
**Proportion of clinical measurements (M) and clinical codes (C) among selected features**.

**Figure 6:**
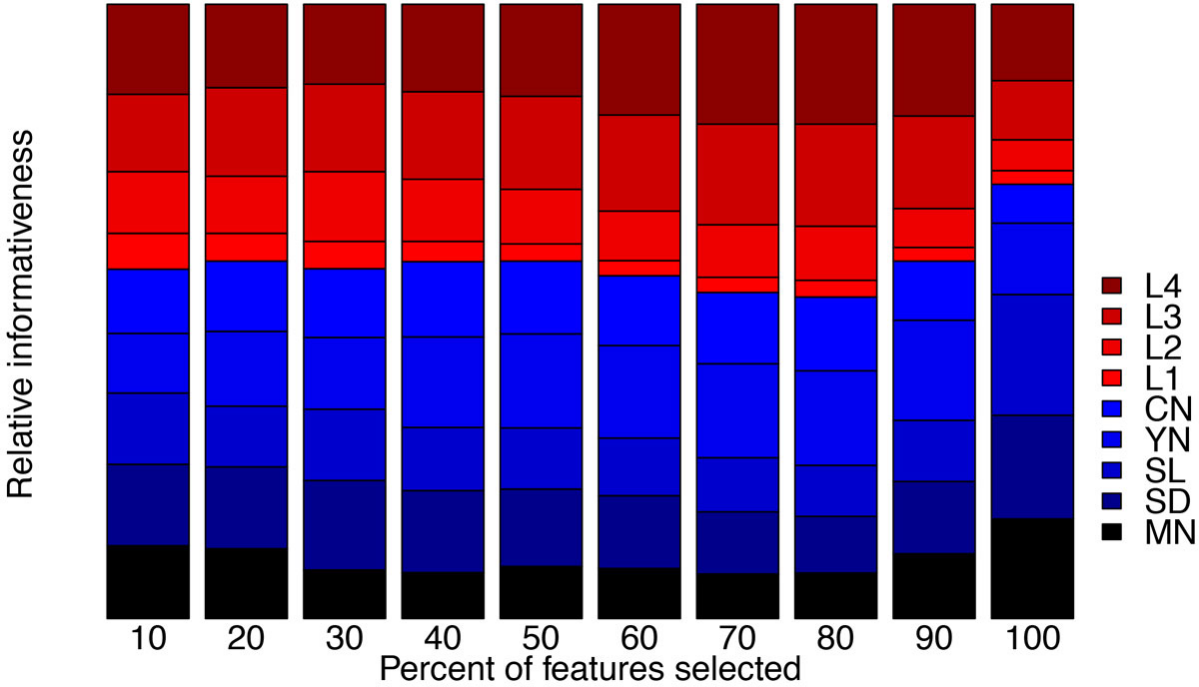
**Relative informativeness of the 5 representations of clinical measurements (MN: mean; SD: standard deviation; SL: slope; YN: existence; CN: count) and 4 levels (L1 - L4) of clinical codes based on their information gain scores**. Larger area indicates lower informativeness.

From a quantitative point of view, the bar plot in Figure 5, depicting the proportion of clinical measurements and clinical codes among the selected features indicates that, irrespective of threshold, the majority are invariably clinical codes. From a qualitative point of view, as shown in Figure 6, the relative informativeness of specific representations of each data type according to their information gain scores tells us that clinical codes are generally more informative than clinical measurements.

**Figure 7:**
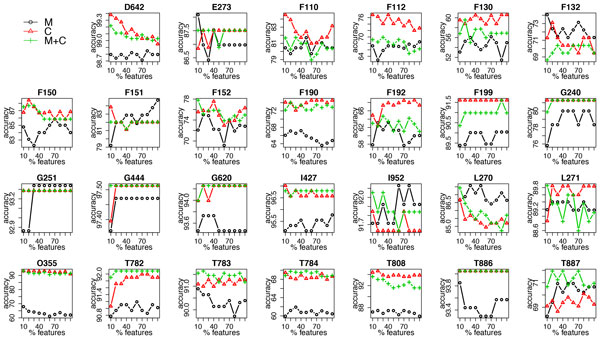
**Accuracy of random forest using clinical measurements (M), clinical codes (C) and their combination (M+C) at each feature selection threshold in each dataset**.

**Figure 8:**
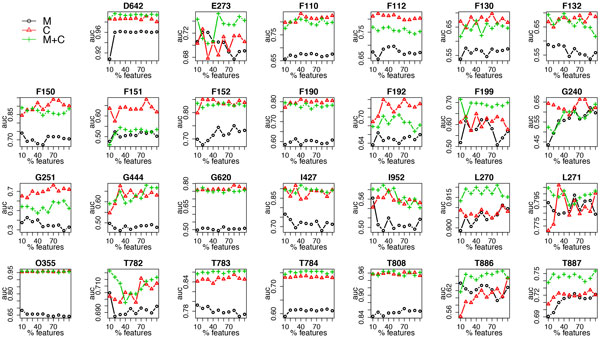
**AUC of random forest using clinical measurements (M), clinical codes (C) and their combination (M+C) at each feature selection threshold in each dataset**.

Moreover, due to the distinct nature of different ADEs, we also present results for each individual dataset in Figure 7 for accuracy and Figure 8 for AUC, respectively. For datasets such as D642, which has the largest number of features, feature selection clearly improves the accuracy; while for some datasets, such as T783 and T887, using a combination of the two data types yields the best predictive performance.

### Using various learning algorithms

**Figure 9:**
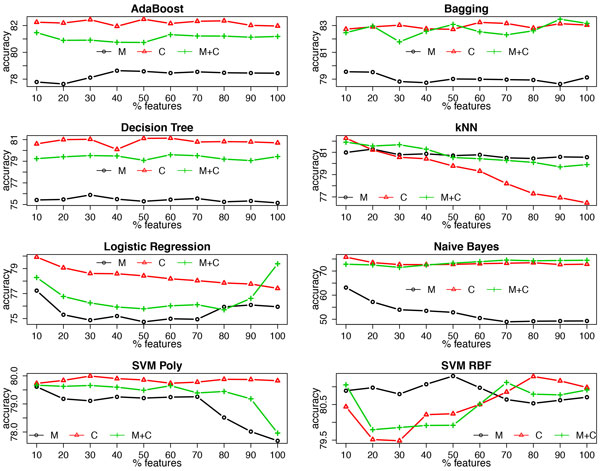
**Accuracy of multiple classifiers using clinical measurements (M), clinical codes (C) and their combination (M+C) at each feature selection threshold**.

**Figure 10:**
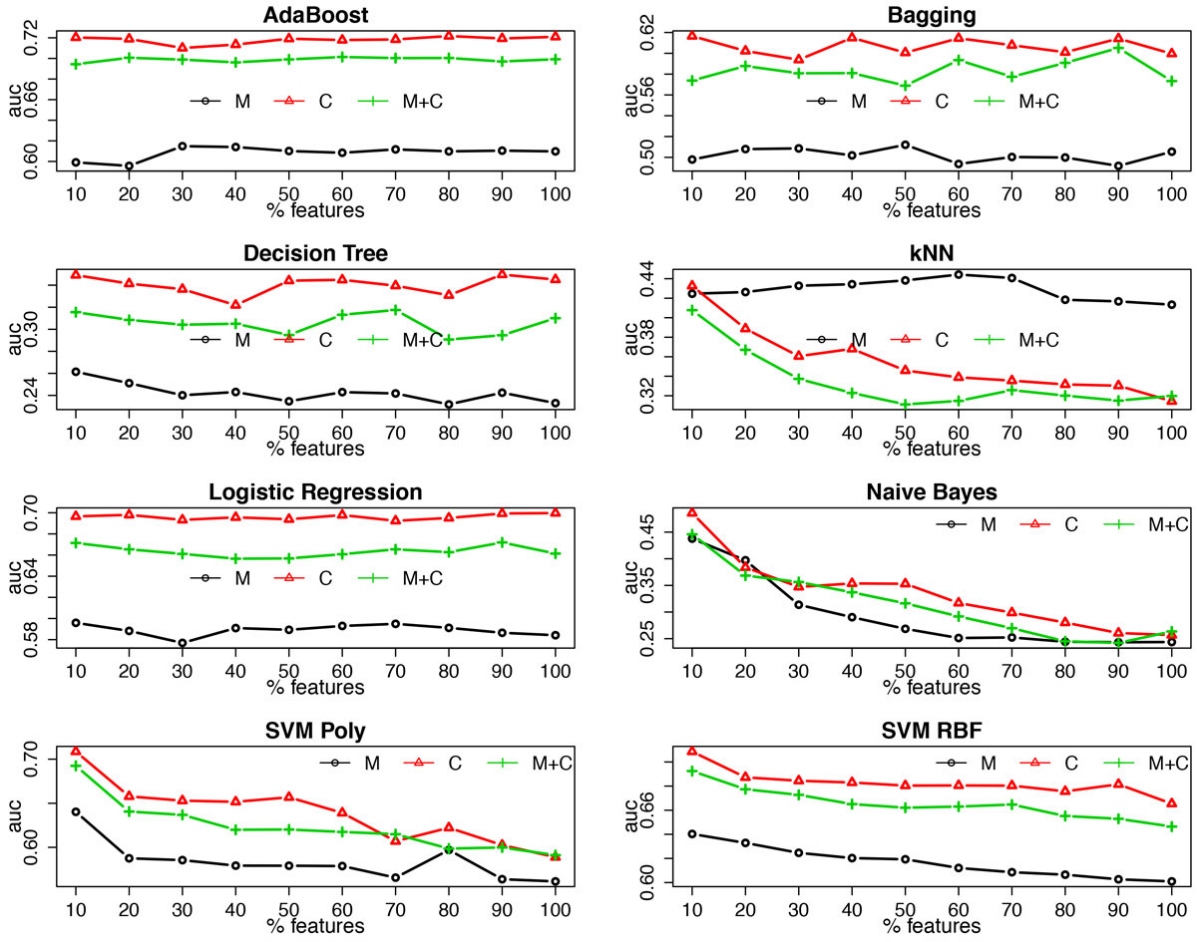
**AUC of multiple classifiers using clinical measurements (M), clinical codes (C) and their combination (M+C) at each feature selection threshold**.

Figure 9 and Figure 10 demonstrate the averaged accuracy and AUC, respectively, of eight additional commonly used learning algorithms using clinical measurements, clinical codes and their combination over the 27 datasets. It is clear that the random forest algorithm outperforms the others; for most learning algorithms, using clinical codes yields the best predictive performance; however, for learning algorithms that are very sensitive to high dimensionality, such as *k *nearest neighbors (kNN), using measurements alone and/or applying feature selection improve(s) the predictive performance, as the number of clinical measurements is far smaller than the number of clinical codes.

## Discussion

This study investigated the use of various types of structured EHR data - clinical measurements and clinical codes - both in isolation and in combination, to build machine learning models for ADE detection. The results show that using clinical codes alone, or together with clinical measurements, leads to significantly improved predictive performance compared to using only clinical measurements. In addition, feature selection based on information gain was conducted to remove relatively less informative variables, which also enables a deeper inspection of the informativeness of each data type and representation.

### Results analysis

We evaluated different representations of clinical measurements and clinical codes using methods proposed in [24] and [26], and slightly different results are observed here. In the previous study that explored the possibility of exploiting the concept hierarchies of clinical codes [26], it was demonstrated that using only the more general levels of the codes was sufficient to maintain the predictive performance on a high level; in this study, however, we observed that using all levels of the codes, including both the general and the more specific levels, yields the best predictive performance. A possible explanation for this is that the tasks in the two studies are different: in [26], the task was to distinguish patients with a specific ADE from randomly selected patients without the ADE; in this study, the task was to distinguish patients with a specific ADE from patients with a similar disease to the ADE. The latter is a much more difficult task than the former, as the positive and negative examples are more similar in the latter. It is thus not surprising that, in this task, more specific levels of codes are needed to improve the predictive performance. In the study that investigates various representations of clinical measurements [24], the model with a combination of multiple representations outperformed the ones with any single representation, which is consistent with the observation in this study; however, the predictive performance of models using the single representations are inconsistent with the previous study: *Mean *is the best in the former, while *Count *is the best in the latter. This discrepancy might be due to slightly different settings of the tasks in the two studies. In [24], the task was also to distinguish patients with a specific ADE and patients with similar diseases to the ADE, but it is achieved by retrospectively analyzing the entire available patient history in the EHRs, i.e., clinical events that occurred after the target ADE were included in the predictive models; in this study, the task was instead designed for detecting ADEs at the point of care, which means that only the clinical events that occurred prior to the target ADE were allowed to be exploited in the predictive models.

By combining clinical measurements and clinical codes, the predictive performance does not outperform using only clinical codes. In order to understand the reasons for this observation, we looked at the number of features selected from each data type and their corresponding relative informativeness by ranking features based on their information gain. In general, most of the selected features are clinical codes, which is partly biased as there are in fact more codes than measurements in the feature set, but even when only the top 10% of features are selected, the majority of the top-ranked features are clinical codes. Since only looking at the quantity is not fair in this case, we instead inspected the relative informativeness, adjusted by the number of features, between codes and measurements. It turned out that clinical codes were consistently more informative than clinical measurements. Although by using only clinical measurements, the predictive performance is not worse than random guessing (average accuracy of 81.41 and AUC of 0.655), adding them to clinical codes does not seem to be helpful in improving the predictive performance compared to using codes alone. This can partly be explained by how each tree is built in the random forest: the algorithm selects the most informative feature from a random subset of features as the node to split on when building each tree. In this case, clinical measurements are less likely to be selected as they are inferior to clinical codes in terms of both quantity and quality. As a result, they can almost be considered useless when used in conjunction with clinical codes.

Besides the random forest algorithm, we also employed several other common learning algorithms. Similar results are observed with AdaBoost, Bagging and decision tree as were observed for the random forest algorithm, while for the other learning algorithms that are neither tree-based nor ensemble models, the results deviate from the previous pattern. For example, logistic regression favors the combination of clinical codes and measurements when no feature selection is conducted; a support vector machine with the RBF kernel using clinical measurements yields better predictive performance when only part of the features are selected; and the *k *nearest neighbor algorithm always achieves better performance by using clinical measurements alone. Moreover, feature selection has a different impact on these learning algorithms, which is basically consistent with what we know about their sensitiveness towards high dimensionality, e.g., adding feature selection clearly improves the predictive performance of the *k *nearest neighbor algorithm. Here, it is worth noting that among all of the investigated learning algorithms, the random forest classifier consistently outperforms the others for this task, which, again, proves its robustness on handling high dimensional data.

In addition to the averaged results over the 27 datasets, we also presented results for each individual dataset. For most datasets, using only clinical measurements results in the worst performance; however, if we look at the results for accuracy, for some datasets, such as G251, F132 and L270, opposite results are observed; for the AUC results, we can see that for datasets D642, E273, F199, L270, T783, T784, T886 and T887, using a combination of clinical measurements and codes outperforms the others. These diverse results can perhaps be explained by the different nature of each ADE. For example, to detect D642 (drug induced anemia), using clinical codes only is probably not sufficient since such a diagnosis is often made after observing results from blood tests; to detect ADEs starting with F (mental and behavioural disorders), it is less likely that using clinical measurements is helpful, whereas clinical notes, in this case, might contain much more valuable information than the structured data.

### Challenges of using electronic health records for adverse drug event detection

Although EHRs are increasingly considered as a valuable resource for pharmocavgilance and machine learning based methods are often favored over other methods when analyzing large amounts of data from EHRs, it is, by using such purely data-driven methods, difficult to distinguish clinically relevant signals from systematic biases in the data. Therefore, the machine learning methods should serve primarily as tools for exploring the massive amounts of data and testing hypotheses; eventually, human knowledge and experience is still necessary to evaluate the validity of the findings.

In addition to the challenges that have already been discussed in the background section, EHR data is also very noisy. On the one hand, the quality of the diagnosis encoding varies according to the experience and expertise of coders [38], making it difficult for data analysts to adjust the validity and reliability of the reported events. According to a review by the Swedish National Board of Health and Welfare, around 20% of the assigned primary diagnosis codes were found to be erroneous [39]. On the other hand, clinical codes can be influenced by various factors, such as the knowledge and experience of the clinicians, the amount of information available at admission and strategic billing, rendering the choice of codes to report biased. In such situations, when the codes are used to label the training data, we should proceed with caution as they cannot entirely be considered as a gold standard. One expensive alternative here is to involve experts for reviewing training data and correcting incorrect labels.

### Limitations and future work

One limitation of this study is that the labels in the training data are directly extracted from the EHR database without being scrutinized by clinical experts. This could lead to findings that do not entirely reflect reality. Moreover, both clinical codes and measurements are represented in certain ways in this study, and hence the results and findings are limited only to these representations. It is, for instance, conceivable that, with better representations, clinical measurements would be as informative as clinical codes for detecting ADEs. Therefore, in future work, representations that can further improve the informativeness of clinical measurements should be explored. This study only included two types of data, codes and measurements, from EHRs. A natural extension would thus be to include more data types, such as lab tests and notes.

## Conclusions

We have here demonstrated how machine learning can be employed to analyze structured data in electronic health records for the purpose of supporting pharmacovigilance activities such as detecting adverse drug events. Predictive models learned from electronic health records could be incorporated into adverse drug event alerting systems at the point of care, primarily facilitating the correct encoding of adverse drug events, which, in turn, would address the problem of under-reporting of adverse drug events and lead to more reliable statistics. To create high-performing predictive models, it is essential to pay careful attention to which data to use and how to best represent it, especially so when faced with high-dimensional and extremely sparse data. We have here presented a detailed study and proposed solutions to the said challenges, focusing on two groups of data: measurements and clinical codes that encode drugs and diagnoses.

Within each data type, it is advantageous to combine multiple representations, effectively providing a more holistic view of the data. Across data types, providing all representations of each data type leads to improved predictive performance for some learning algorithms, while for the best-performing learning algorithm - random forest - this is beneficial in certain cases only, i.e., for specific adverse drug events. Generally speaking, clinical codes are more informative than measurements for the purpose of detecting adverse drug events, and it is not necessary in general to add measurements to clinical codes. Selecting a subset of the most informative features can, to some extent, lead to improved predictive performance, even with learning algorithms that are considered to effectively handle high-dimensional data.

## Competing interests

The authors declare that they have no competing interests.

## Authors' contributions

JZ, AH, LA and HB were involved in discussions on the study design. JZ and AH conducted the experiments. JZ created a draft of the manuscript. AH and HB commented on the draft. JZ, AH, LA and HB proofread the manuscript.

## Declarations

Publication costs for this article were funded by the project High-Performance Data Mining for Drug Effect Detection at Stockholm University.

This article has been published as part of *BMC Medical Informatics and Decision Making *Volume 15 Supplement 4, 2015: Selected articles from the IEEE International Conference on Bioinformatics and Biomedicine (BIBM 2014): Medical Informatics and Decision Making. The full contents of the supplement are available online at http://www.biomedcentral.com/bmcmedinformdecismak/supplements/15/S4.

## References

[B1] Classen DC Resar R Griffin F Federico F Frankel T Kimmel N Whittington JC Frankel A Seger A James BC 'Global trigger tool' shows that adverse events in hospitals may be ten times greater than previously measured Health Affairs 2011 30 4 581 589 21471476 10.1377/hlthaff.2011.0190

[B2] Hakkarainen KM Hedna K Petzold M Hägg S Percentage of patients with preventable adverse drug reactions and preventability of adverse drug reactions-a meta-analysis PloS One 2012 7 3 33236 10.1371/journal.pone.0033236 PMC3305295 22438900

[B3] Beijer H De Blaey C Hospitalisations caused by adverse drug reactions (adr): a meta-analysis of observational studies Pharmacy World and Science 2002 24 2 46 54 12061133 10.1023/a:1015570104121

[B4] Nebeker JR Hoffman JM Weir CR Bennett CL Hurdle JF High rates of adverse drug events in a highly computerized hospital Archives of internal medicine 2005 165 10 1111 1116 15911723 10.1001/archinte.165.10.1111

[B5] Organization WH The importance of pharmacovigilance 2002

[B6] Härmark L Van Grootheest A Pharmacovigilance: methods, recent developments and future perspectives European Journal of Clinical Pharmacology 2008 64 8 743 752 18523760 10.1007/s00228-008-0475-9

[B7] Ahmad SR Adverse drug event monitoring at the food and drug administration Journal of general internal medicine 2003 18 1 57 60 12534765 10.1046/j.1525-1497.2003.20130.x PMC1494803

[B8] Lindquist M Vigibase, the who global icsr database system: basic facts Drug Information Journal 2008 42 5 409 419

[B9] Hazell L Shakir SA Under-reporting of adverse drug reactions Drug Safety 2006 29 5 385 396 16689555 10.2165/00002018-200629050-00003

[B10] Goldman SA Limitations and strengths of spontaneous reports data Clinical Therapeutics 1998 20 40 44 9915089 10.1016/s0149-2918(98)80007-6

[B11] Trifirò G Patadia V Schuemie MJ Coloma PM Gini R Herings R Hippisley-Cox J Mazzaglia G Giaquinto C Scotti L EU-ADR healthcare database network vs. spontaneous reporting system database: preliminary comparison of signal detection Studies in Health Technology and Informatics 2011 166 25 30 21685607

[B12] Kohane IS Using electronic health records to drive discovery in disease genomics Nature Reviews Genetics 2011 12 6 417 428 10.1038/nrg2999 21587298

[B13] Roque FS Jensen PB Schmock H Dalgaard M Andreatta M Hansen T Søeby K Bredkjær S Juul A Werge T Using electronic patient records to discover disease correlations and stratify patient cohorts PLoS Computational Biology 2011 7 8 1002141 10.1371/journal.pcbi.1002141 PMC3161904 21901084

[B14] Jensen PB Jensen LJ Brunak S Mining electronic health records: towards better research applications and clinical care Nature Reviews Genetics 2012 13 6 395 405 10.1038/nrg3208 22549152

[B15] Bishop CM Pattern Recognition and Machine Learning 2006 4 Springer, New York

[B16] Larrañaga P Calvo B Santana R Bielza C Galdiano J Inza I Lozano JA Armañanzas R Pérez A Machine learning in bioinformatics Briefings in Bioinformatics 2006 7 1 86 112 16761367 10.1093/bib/bbk007

[B17] Hauben M Madigan D Gerrits CM Walsh L Van Puijenbroek EP The role of data mining in pharmacovigilance Expert Opinion on Drug Safety 2005 10.1517/14740338.4.5.929 16111454

[B18] Harpaz R DuMouchel W Shah NH Madigan D Ryan P Friedman C Novel data-mining methodologies for adverse drug event discovery and analysis Clinical Pharmacology & Therapeutics 2012 91 6 1010 1021 22549283 10.1038/clpt.2012.50 PMC3675775

[B19] LePendu P Iyer SV Bauer-Mehren A Harpaz R Mortensen JM Podchiyska T Ferris TA Shah NH Pharmacovigilance using clinical notes Clinical Pharmacology & Therapeutics 2013 93 6 547 555 23571773 10.1038/clpt.2013.47 PMC3846296

[B20] Eriksson R Jensen PB Frankild S Jensen LJ Brunak S Dictionary construction and identification of possible adverse drug events in danish clinical narrative text JAMIA 2013 20 5 947 953 23703825 10.1136/amiajnl-2013-001708 PMC3756275

[B21] Henriksson A Kvist M Hassel M Dalianis H Exploration of adverse drug reactions in semantic vector space models of clinical text Proceedings of ICML Workshop on Machine Learning for Clinical Data Analysis 2012

[B22] Batal I Fradkin D Harrison J Moerchen F Hauskrecht M Mining recent temporal patterns for event detection in multivariate time series data Proceedings of the 18th ACM SIGKDD International Conference on Knowledge Discovery and Data Mining 2012 ACM 280 288 10.1145/2339530.2339578 PMC4414327 25937993

[B23] Chazard E Ficheur G Bernonville S Luyckx M Beuscart R Data mining to generate adverse drug events detection rules Information Technology in Biomedicine, IEEE Transactions 2011 15 6 823 830 10.1109/TITB.2011.2165727 21859604

[B24] Zhao J Henriksson A Asker L Boström H Detecting adverse drug events with multiple representations of clinical measurements Proceedings of International Conference on Bioinformatics and Biomedicine: 2-5 November 2014 2014 Belfast, UK, IEEE Computer Society 536 543

[B25] Karlsson I Zhao J Asker L Boström H Predicting adverse drug events by analyzing electronic patient records Proceedings of Conference on Artificial Intelligence in Medicine 2013 Springer 125 129

[B26] Zhao J Henriksson A Boströom H Detecting adverse drug events using concept hierarchies of clinical codes Proceedings of International Conference on Healthcare Informatics 2014 IEEE Computer Society 285 293

[B27] Ficheur G Chazard E Beuscart J-B Merlin B Luyckx M Beuscart R Adverse drug events with hyperkalaemia during inpatient stays: evaluation of an automated method for retrospective detection in hospital databases BMC Medical Informatics and Decision Making 2014 14 1 83 25212108 10.1186/1472-6947-14-83 PMC4164763

[B28] Dalianis H Hassel M Henriksson A Skeppstedt M Stockholm epr corpus: a clinical database used to improve health care Swedish Language Technology Conference 2012 17 18

[B29] Stausberg J Hasford J Drug-related admissions and hospital-acquired adverse drug events in germany: a longitudinal analysis from 2003 to 2007 of icd-10-coded routine data BMC Health Services Research 2011 11 1 134 21619706 10.1186/1472-6963-11-134 PMC3116475

[B30] Breiman L Random forests Machine Learning 2011 45 1 5 32

[B31] Caruana R Karampatziakis N Yessenalina A An empirical evaluation of supervised learning in high dimensions Proceedings of the 25th International Conference on Machine Learning 2008 ACM 96 103

[B32] Bradley AP The use of the area under the roc curve in the evaluation of machine learning algorithms Pattern Recognition 1997 30 7 1145 1159

[B33] Demšar J Statistical comparisons of classifiers over multiple data sets The Journal of Machine Learning Research 2006 7 1 30

[B34] Garcia S Herrera F An extension on "statistical comparisons of classifiers over multiple data sets" for all pairwise comparisons Journal of Machine Learning Research 2008 9 12

[B35] Boström H Feature vs. classifier fusion for predictive data mining a case study in pesticide classification Proceedings of the 10th International Conference on Information Fusion 2007 IEEE 1 7

[B36] Kohavi R John GH Wrappers for feature subset selection Artificial Intelligence 1997 97 1 273 324

[B37] Lazar C Taminau J Meganck S Steenhoff D Coletta A Molter C de Schaetzen V Duque R Bersini H Nowe A A survey on filter techniques for feature selection in gene expression microarray analysis IEEE/ACM Transactions on Computational Biology and Bioinformatics (TCBB) 2012 9 4 1106 1119 10.1109/TCBB.2012.33 22350210

[B38] Puentes J Montagner J Lecornu L Cauvin J-M Information quality measurement of medical encoding support based on usability Computer methods and programs in biomedicine 2013 112 3 329 342 23958646 10.1016/j.cmpb.2013.07.018

[B39] Socialstyrelsen: The National Board of Health and Welfare, Diagnosgranskningar utförda i Sverige 1997-2005 samt råd inför granskning, (In Swedish) 2006 http://www.socialstyrelsen.se/publikationer2006/2006-131-30

